# Antimicrobial and Antibiofilm Effects of Combinatorial Treatment Formulations of Anti-Inflammatory Drugs—Common Antibiotics against Pathogenic Bacteria

**DOI:** 10.3390/pharmaceutics15010004

**Published:** 2022-12-20

**Authors:** Fatemehalsadat Tabatabaeifar, Elham Isaei, Davood Kalantar-Neyestanaki, José Rubén Morones-Ramírez

**Affiliations:** 1 Facultad de Ciencias Químicas, Universidad Autónoma de Nuevo León, UANL, Av. Universidad S/N, San Nicolás de los Garza 66455, Mexico; 2 Centro de Investigación en Biotecnología y Nanotecnología, Facultad de Ciencias Químicas, Universidad Autónoma de Nuevo León, Parque de Investigación e Innovación Tecnológica, Km. 10 Autopista al Aeropuerto Internacional Mariano Escobedo, Apodaca 66629, Mexico; 3 Student Research Committee, Kerman University of Medical Sciences, Kerman 7616913555, Iran; 4 Medical Mycology and Bacteriology Research Center, Kerman University of Medical Sciences, Kerman 7616913555, Iran; 5 Department of Medical Microbiology (Bacteriology & Virology), Afzalipour Faculty of Medicine, Kerman University of Medical Sciences, Kerman 7616913555, Iran

**Keywords:** antibiotics, biofilm, anti-inflammatory drugs, *icaA*, *algD*

## Abstract

With the spread of multi-drug-resistant (MDR) bacteria and the lack of effective antibiotics to treat them, developing new therapeutic methods and strategies is essential. In this study, we evaluated the antibacterial and antibiofilm activity of different formulations composed of ibuprofen (IBP), acetylsalicylic acid (ASA), and dexamethasone sodium phosphate (DXP) in combination with ciprofloxacin (CIP), gentamicin (GEN), cefepime (FEP), imipenem (IPM), and meropenem (MEM) on clinical isolates of *Staphylococcus aureus* (*S. aureus*) and *Pseudomonas aeruginosa* (*P. aeruginosa*) as well as the transcription levels of biofilm-associated genes in the presence of sub-MICs of IBP, ASA, and DXP. The minimal inhibitory concentrations (MICs), minimal biofilm inhibitory concentrations (MBICs), and minimum biofilm eradication concentrations (MBECs) of CIP, GEN, FEP, IPM, and MEM with/without sub-MICs of IBP (200 µg/mL), ASA (200 µg/mL), and DXP (500 µg/mL) for the clinical isolates were determined by the microbroth dilution method. Quantitative real-time-PCR (qPCR) was used to determine the expression levels of biofilm-related genes, including *icaA* in *S. aureus* and *algD* in *P. aeruginosa* at sub-MICs of IBP, ASA, and DXP. All *S. aureus* isolates were methicillin-resistant *S. aureus* (MRSA), and all *P. aeruginosa* were resistant to carbapenems. IBP decreased the levels of MIC, MBIC, and MBEC for all antibiotic agents in both clinical isolates, except for FEP among *P. aeruginosa* isolates. In MRSA isolates, ASA decreased the MICs of GEN, FEP, and IPM and the MBICs of IPM and MEM. In *P. aeruginosa*, ASA decreased the MICs of FEP, IPM, and MEM, the MBICs of FEP and MEM, and the MBEC of FEP. DXP increased the MICs of CIP, GEN, and FEP, and the MBICs of CIP, GEN, and FEP among both clinical isolates. The MBECs of CIP and FEP for MRSA isolates and the MBECs of CIP, GEN, and MEM among *P. aeruginosa* isolates increased in the presence of DXP. IBP and ASA at 200 µg/mL significantly decreased the transcription level of *algD* in *P. aeruginosa*, and IBP significantly decreased the transcription level of *icaA* in *S. aureus*. DXP at 500 µg/mL significantly increased the expression levels of *algD* and *icaA* genes in *S. aureus* and *P. aeruginosa* isolates, respectively. Our findings showed that the formulations containing ASA and IBP have significant effects on decreasing the MIC, MBIC, and MBEC levels of some antibiotics and can down-regulate the expression of biofilm-related genes such as *icaA* and *algD*. Therefore, NSAIDs represent appropriate candidates for the design of new antibacterial and antibiofilm therapeutic formulations.

## 1. Introduction

With the increased antibiotic resistance among bacteria, we are getting closer to re-living the pre-antibiotic era. Moreover, the slow rate of current antibiotic discovery might lead society to experience, in the near future, a shortage of effective antibiotic treatments to treat bacterial infections caused by resistant microorganisms. Therefore, developing new approaches and strategies is essential for treating infections caused by resistant bacteria [1]. Several studies have shown the antimicrobial and antibiofilm activity of nonsteroidal anti-inflammatory drugs (NSAIDs) and we have different reports about the synergistic effects of NSAIDs with antibacterial agents [1,2]. Moreover, some studies have shown antagonistic interactions between antibiotics with anti-inflammatory drugs and an increase in the MICs of antibiotics agents as well as an increasing biofilm formation in the presence of anti-inflammatory drugs, especially corticosteroids such as dexamethasone [1,2]. Anti-inflammatory drugs are divided into two groups: corticosteroids and NSAIDs [3]. These drugs are widely used in combination with antibiotics to treat infections since they are administered to reduce inflammation, pain, and fever [3]. The mechanism of action of NSAIDs such as ibuprofen and acetylsalicylic acid (aspirin) depends on the inhibition of cyclooxygenase enzymes [3]. Corticosteroid drugs such as dexamethasone and betamethasone are synthetic analogs of glucocorticoids, which have anti-inflammatory and immunosuppressive functions and their effects depend on blocking receptors and genomic and nongenomic pathways [3]. Bacteria can develop resistance to antibiotics through the formation of biofilms, acquisition of resistance genes, and chromosomal mutations [4,5]. In addition to developing resistance to antibiotics, biofilm formation in bacteria is an important defense mechanism against the host immune system, leading to recurrent infections and increasing the pathogenicity of bacteria [6]. Moreover, there is a strong correlation between bacterial biofilm formation and the development of infective endocarditis, atherosclerosis, and urinary tract infections [7]. *Staphylococcus aureus* (*S. aureus*) and *Pseudomonas aeruginosa* (*P. aeruginosa*) are important opportunistic human pathogens capable of causing severe infections in hospital settings, especially in immunosuppressed patients [8,9]. Biofilm formation in these bacteria plays an important role in their pathogenicity and resistance to antibiotic agents [6]. The *algD* operon in *P. aeruginosa* encodes the main enzymes for alginate biosynthesis. Alginate is a homo- and hetero-polysaccharide that consists of β-D-mannuronate (M) and α-L-guluronate (G) units and plays an important role in biofilm formation in *P. aeruginosa* [5,6]. In *S. aureus,* the *icaABCD* operon plays an important role in biofilm formation [10]. It has been demonstrated that biofilm formation in *Staphylococcus* spp. is mediated by the *ica* operon. This operon consists of the four genes, *icaA*, *icaD*, *icaB*, and *icaC,* that appear to be negatively regulated by *icaR* [10].

Several studies have shown the antibacterial effects of NSAIDs such as ibuprofen, diclofenac, aspirin, and celecoxib against various bacteria such as *Klebsiella pneumoniae*, *Escherichia coli*, *P. aeruginosa*, and *S. aureus* [1,11]. NSAIDs, in addition to antibacterial effects in the combination of antibiotics, increase the susceptibility of bacteria to antibiotics agents [1]. Therefore, combining antibiotic agents with NSAIDs can be a strategy for treating bacterial infections and controlling inflammatory conditions.

Imipenem, meropenem, cefepime, ciprofloxacin, and gentamicin are among the most widely used antibiotics in the treatment of various bacterial infections especially in hospital settings; therefore, due to the simultaneous use of these antibiotics agents with anti-inflammatory drugs in treating infections, it is essential to pay attention to the interactions between these drugs when administered simultaneously. Moreover, increasing our knowledge in this regard could help us to uncover new strategies and methods for treating infections. In the present study, we determined the antibacterial and antibiofilm effects of treatment formulations composed of commonly used NSAIDs including ibuprofen (IBP), aspirin (acetylsalicylic acid: ASA), and dexamethasone sodium phosphate (DXP) in combination with ciprofloxacin, gentamicin, cefepime, imipenem, and meropenem as current antimicrobial agents; moreover, we monitored the transcriptional levels of biofilm-involved genes, including *icaA* and *algD* in *S. aureus* and *P. aeruginosa*, respectively, at sub-MICs of IBP, ASA, and DXP.

## 2. Materials and Methods

### 2.1. Bacterial Isolates

Bacterial isolates, including *S. aureus* (n = 10) and *P. aeruginosa* (n = 10), were collected from various clinical samples in Afzalipour hospital in Kerman, Iran, and the San Bernardo Hospital in Monterrey, Nuevo León, Mexico. All bacteria were confirmed and identified by biochemical and standard microbiological tests [12,13,14].

### 2.2. Antibacterial Susceptibility of Isolates

At first, 0.5 McFarland’s concentration was prepared from each of the clinical isolates to perform antibiotic susceptibility tests to different antibiotic agents according to the Clinical & Laboratory Standards Institute (CLSI) recommendations for the disk diffusion method on Mueller Hinton Agar (MHA; Condalab Co., Madrid, Spain) [15]. The following antibiotic disks, including penicillin (P, 10 units), gentamicin (GEN, 10 μg), amikacin (AM, 30 μg), erythromycin (E, 15 μg), ciprofloxacin (CIP, 5 μg), tetracycline (TE, 30 μg), clindamycin (CL, 2 μg) trimethoprim/sulfamethoxazole (SXT, 1.25/23.75μg), and linezolid (LIN, 30 µg) (Mast Group Ltd., Liverpool, UK), were used for *S. aureus* isolates in the disk diffusion method and meropenem (MEM, 10 μg), doripenem (DOR, 10 μg), imipenem (IPM, 10 μg), ciprofloxacin (CIP, 5 μg), piperacillin/tazobactam (PTZ, 100/10 μg), gentamicin (GEN, 10 μg), aztreonam (AZT, 30 μg), cefepime (FEP, 10 μg), and ceftazidime (CAZ, 30 μg) disks (Mast Group Ltd., Liverpool, U.K.) were used for *P. aeruginosa* isolates. Methicillin-resistant *Staphylococcus aureus* (MRSA) isolates were screened using a cefoxitin (FOX, 30 μg) disk. According to the CLSI recommendations, the microbroth dilution method was used to determine the minimum inhibitory concentrations (MICs) of GEN, CIP, FEP, IPM, MEM (Sigma-Aldrich, Inc., St. Louis, MO 68178, USA), aspirin (ASA; acetylsalicylic acid, Temad Pharmaceutical, Co, Tehran, Iran), ibuprofen (IBP; Temad Pharmaceutical, Co, Tehran, Iran), and dexamethasone sodium phosphate (DXP; Sinadarou Pharmaceutical, Co, Tehran, Iran) for both clinical isolates. for The MIC experiments were performed in triplicate and *S. aureus* ATCC 25923 and *P. aeruginosa* ATCC 27853 were used as control strains in antibacterial susceptibility tests.

### 2.3. DNA Extraction and Detection of mecA and icaADBC Operon in S. aureus Isolates and algD in P. aeruginosa

The total DNA of the isolates was extracted by the boiling methods previously described [16]. Briefly, 3–4 bacterial colonies from pure cultures of clinical isolates on brain heart infusion agar (BHI, Condalab Co., Madrid, Spain) were removed and suspended in 500 µL sterile DNAase and RNAase free water and boiled for 5 min and centrifuged at 10,000 rpm. Then, the supernatant was used as DNA template for amplification in the polymerase chain reaction (PCR) technique. The PCR method was used for the detection of *mecA* and the *icaADBC* operon in *S. aureus* and *algD* in *P. aeruginosa* isolates [13,17,18]. The PCR conditions and primer sequences used to detect *mecA*, *icaADBC*, and *algD* are presented in Table 1. A PCR technique was performed in a volume of 25 µL, containing the following: 12.5 µL of Taq DNA Polymerase Master Mix RED (Ampliqon, Co., Stenhuggervej, Denmark), 1 µL of DNA template, 0.25 µL of each forward and reverse primer (10 pM), and 11 µL sterile DNase and RNase free water. The PCR products were evaluated by electrophoresis on 1% agarose gel in 0.5 × TBE buffer (5.4 g Tris base, 2.75 g Boric acid, 2 mL 0.5 M EDTA, in 1 L sterile water) and stained by Green Viewer dye (Green Viewer, Parstous Biotechnology, Co., Mashhad, Iran), and a gel image was obtained by using a gel documentation system.

### 2.4. Biofilm Production Assay

For the biofilm formation assay among clinical isolates of *S. aureus* and *P. aeruginosa*, we used the microtiter method previously described by Stepanovic et al. using 96-well polystyrene microtiter sterile plates [19]. Briefly, clinical isolates were cultured on nutrient agar plates at 37 °C overnight, and 0.5 McFarland standard was prepared from each bacterial sample in phosphate-buffered saline (PBS). Then, 20 μL of bacterial suspension was mixed with 180 μL trypticase soy broth (TSB, Condalab Co., Madrid, Spain) supplemented with 1% glucose and added to sterile 96-well polystyrene microtiter plates. After incubation overnight at 37 °C, the microplates were carefully washed three times with sterile PBS, and then microplates were inverted to dry for 20 min at room temperature. For biofilm quantification, 200 μL of 2% safranin dye solution was added to the well for 40 min at room temperature and washed three times with sterile PBS. Safranin bound to the biofilm in each well was extracted with 200 μL of pure ethanol, and the absorbance of the extracted safranin was measured at 490 nm in an ELISA reader (BioTek, Agilent Technologies, Inc., Santa Clara, CA 95051, USA). Each assay was performed in triplicate. TSB + 1% glucose medium was used as a negative control to determine background optical density (OD). The cut-off ODs for biofilm formation were determined as the average OD of the negative control + 3 × standard deviation (SD) of the negative control. The OD value was calculated for each microtiter plate separately. OD > 4 × ODc was considered a strong biofilm formation; 2 × ODc < OD ≤ 4 × ODc was considered a moderate biofilm formation; ODc < OD ≤ 2 × ODc was considered a weak biofilm formation; and OD ≤ ODc was considered a nonbiofilm formation. *Staphylococcus epidermidis* RP62A (ATCC 35984) and *Pseudomonas aeruginosa* PAO1 were used as positive controls in biofilm formation assays.

### 2.5. Anti-Inflammatory Drugs Interference Experiments

To evaluate the possible effect of anti-inflammatory drugs, including ASA, IBP, and DXP, on the antibacterial and antibiofilm activity of antibiotic agents, we determined the MIC, minimum biofilm inhibitory concentration (MBIC), and minimum biofilm eradication concentration (MBEC) of each antibiotic agent including GEN, CIP, FEP, IPM, and MEM with/without sub-MIC ASA, IBP, and DXP. DMSO was used as a solvent for anti-inflammatory drugs, and 10 mg/mL stock solutions of IBP, ASA, and DXP in DMSO were used to prepare working solutions of ASA (200 µg/mL), IBP (200 µg/mL), and DXP (500 µg/mL) in an MH broth containing DMSO (5%, vol/vol). MIC, MBIC, and MBEC experiments were performed in triplicate in Mueller Hinton Broth (MH Broth, Condalab, Co, Madrid, Spain), and MH broth with 5% DMSO without IBP, ASA, DXP, and antibiotics was used as a control for the experiment [20]. *Staphylococcus epidermidis* RP62A (ATCC 35984) and *P. aeruginosa* PAO1 were standard strains in interference experiments.

#### 2.5.1. MICs of Isolates to Antibiotics Agents with/without ASA, IBP, and DXP

The MICs of GEN, CIP, FEP, IPM, and MEM for isolates were determined by the microbroth dilution method according to CLSI recommendation with/without 200, 200, and 500 µg/mL of ASA, IBP, and DXP, respectively [15].

#### 2.5.2. MBICs of Antibiotics Agents with/without ASA, IBP, and DXP for Isolates

MBIC assays were performed by the broth microdilution method in 96-well polystyrene sterile plates with a flat-bottom microplate format according to CLSI recommendation. Briefly, a bacterial isolate suspension with an inoculum of 1  ×  10^6^ CFU/mL was diluted in Tryptic Soy Broth (TSB) (Condalab, Co, Madrid, Spain) plus 1% glucose with a serial dilution of antibiotic agents including GEN, CIP, FEP, IPM, and MEM (Sigma-Aldrich, Inc, St. Louis, MO 68178, USA), with/without a constant concentration of one of the sub-MICs of ASA (200 µg/mL), IBP (200 µg/mL), and DXP (500 µg/mL), and then incubated for 24 h at 37 °C. After incubation, MBIC was determined by crystal violet staining as the lowest concentration of antibiotic agents with/without anti-inflammatory drugs that resulted in an OD_600_ difference at or below 10% of the mean of two positive growth-control well readings [21,22].

#### 2.5.3. MBEC of Isolates to Antibiotics Agents with/without ASA, IBP, and DXP

In this step, the effects of each anti-inflammatory drug, along with GEN, CIP, FEP, IPM, and MEM on biofilm eradication were investigated. MBEC assay was performed as previously described [2,21,22]. Biofilms in clinical isolates of *S. aureus* and *P. aeruginosa* were formed overnight in TSB plus 1% glucose at 37 °C in nontreated 96-well polystyrene sterile flat-bottom plates. Biofilms were washed three times with sterile PBS buffer and exposed to different serial dilutions of antibacterial agents, including GEN, CIP, FEP, IPM, and MEM with/without sub-MICs of ASA (200 µg/mL), IBP (200 µg/mL), and DXP (500 µg/mL) in fresh Mueller Hinton Broth. Briefly, the antimicrobial drugs were diluted in fresh MHB, and 50 μL was dispensed in each biofilm in the wells. Stock solutions of ASA, IBP, and DXP were prepared in 5% DMSO and diluted in sterile MHB to reach concentrations of 400, 400, and 1000 μg/mL, respectively, and then 50µL of one of the anti-inflammatory drugs was added to each well with/without the serial dilution of antibiotic agents and plates were incubated for 18 h at 37 °C. The concentration of antibiotic agents with/without IBP, ASA, and DXP that eradicated the mature biofilm was considered the MBEC (Similar to MBIC determination in the Section 2.5.2).

### 2.6. Gene Expression Experiment

Quantitative real-time PCR (qPCR) was used to determine the transcription levels of *icaA* in *S. aureus* and *algD* in *P. aeruginosa* [23,24]. The expression levels of *gyrB* in *S. aureus* and *rpoD* in *P. aeruginosa* were used as reference genes for normalizing the transcriptional levels of *icaA* and *algD* as target genes in *S. aureus* and *P. aeruginosa*, respectively [23,24]. Biofilm gene expression was calculated in each bacterium without the presence (as a calibrator) of anti-inflammatory drugs and with the presence (as treated) of anti-inflammatory drugs. Briefly, clinical isolates were grown separately in MH Broth with ASA (200 µg/mL), IBP (200 µg/mL), and DXP (500 µg/mL) (used as treated isolates) and without anti-inflammatory drugs (used as calibrator isolates) by using a shaker incubator at 37 °C and 180 rpm to the log phase (optical density at 600 nm [OD 600] = 0.8–1) and then bacterial cells were collected by centrifugation at 12,000 rpm in 5 min. The total RNA of bacterial isolates was extracted with RNX-Plus (SINACLON, Co, Tehran, Iran) according to the manufacturer’s recommendations. Then, RNase-Free DNase I enzyme (SINACLON, Co, Tehran, Iran) was used to eliminate DNA contaminations. Total RNA concentration was determined by a Lambda spectrophotometer (PCRmax Limited, Co., Staffordshire, UK), and cDNA synthesis was performed by the Easy cDNA Synthesis Kit (Parstous Biotechnology, Co., Mashhad, Iran), according to the manufacturer’s recommendations. The qPCR experiment was performed in triplicate in the presence of negative and positive controls in a volume of 20 µL, containing the following: 10 µL of Taq DNA Polymerase Master Mix Green (Ampliqon, Co, Stenhuggervej, Denmark), 0.5 µL of cDNA template, 0.2 µL of each primer (10 pM), and 9.1 µL sterile DNase and RNase free water in the StepOnePlus Real-Time PCR System (ThermoFisher SCIENTIFIC, Applied Biosystems, Co., California, USA). qPCR conditions and primer sequences are presented in Table 1. Transcript levels of *icaA* and *algD* were determined relative to the reference genes, and results are expressed as mean values ± standard deviation using a two-sided Student’s t-test and ANOVA tests by GraphPad Prism 8 (GraphPad Software Inc., San Diego, CA, USA) [23,24].

**Table 1:** PCR primer sequences and PCR condition for *mecA* and *ica* genes in this study.

Gene Target	Sequence of Primer (5′-3′)	Product Size (bp)	Annealing (°C)	PCR and qPCR Conditions	Use	Reference
*mecA*	F-TCCAGATTACAACTTCACCAGG R-CCACTTCATATCTTGTAACG	162	56	5 min at 95 °C (1 min at 95 °C, 1 min at annealing temperature, and 1 min at 72 °C for 30 cycles) and 10 min at 72 °C.	In PCR	[13]
*icaA*	F-TCTCTTGCAGGAGCAATCAA R-TCAGGCACTAACATCCAGCA	188	60			[17]
*icaB*	F-ATGGCTTAAAGCACACGACGC R-TATCGGCATCTGGTGTGACAG	526	61			
*icaC*	R-CTCTCTTAACATCATTCCGACGCC F-ATCATCGTGACACACTTACTAACG	1013	63			
*icaD*	F-GAACCGCTTGCCATGTGTTG R-GCTTGACCATGTTGCGTAACC	483	61			
*algD*	F-GCGACCTGGACCTGGGCT R-TTGTGGTCCTGGCAGA	457	56			[18]
*gyrB*	F-AGGTCTTGGAGAAATGAATG R-CAAATGTTTGGTCCGCTT	113	60	15 min at 95 °C (30 s at 95 °C, 30 s at annealing temperature, and 30 s at 72 °C for 40 cycles) and then performed melting curve analysis ranging from 60 to 95 °C	In qPCR for *S. aureus*	[23]
*icaA*	F-AGTTGTCGACGTTGGCTAC R-CCAAAGACCTCCCAATGT	148				
*rpoD*	F-GGGCGAAGAAGGAAATGGTC R-CAGGTGGCCTAGGTGGAGA	178	60		In qPCR for *P. aeruginosa*	[24]
*algD*	F-CGCCGAGATGATCAAGTAC R-TGTAGTAGCGCGACAGGTT	157				

### 2.7. Statistical Analysis

GraphPad Prism 8 (GraphPad Software Inc., USA) was used for the statistical analysis of data and figure production. All data were first assessed for normality using a Kolmogorov–Smirnov test. The results were found to be normally distributed (*p* > 0.05 in the K-S test) and were analyzed using a one-way ANOVA test and expressed as mean values ± standard (mean ± SEM). Pairwise comparisons between groups were then made using Tukey’s post hoc tests, where the main effect was seen in ANOVA tests. Data that were not normally distributed (*p* < 0.05 in the K-S test) were analyzed using a Kruskal–Wallis test. Where the main effect was seen in Kruskal–Wallis tests, pairwise comparisons between groups were made using Dunn’s multiple comparisons test. In each case, *p* < 0.05 was considered statistically significant.

## 3. Results

Clinical isolates of *S. aureus* were resistant to penicillin, gentamicin, amikacin, erythromycin, ciprofloxacin, tetracycline, clindamycin, and trimethoprim/sulfamethoxazole and were sensitive to linezolid and vancomycin. *S. aureus* isolates were MRSA and were positive for *mecA* and the *icaADBC* operon. All clinical isolates of *P. aeruginosa* were resistant to meropenem, doripenem, imipenem, ciprofloxacin, piperacillin/tazobactam, gentamicin, aztreonam, cefepime, and ceftazidime and positive for *algD*. Both clinical isolates were considered biofilm producers according to the microtiter method results. The range of MIC for IBP was 1024–2048 µg/mL, and the MIC range for ASA was 2048–8192 µg/mL among both clinical isolates of *S. aureus* and *P. aeruginosa* (Supplementary Tables S1–S5). DXP had no antibacterial effect against the bacterial isolates. The MIC_50_,_90_, MBIC_50_,_90_, and MBEC_50_, _90_ of antibiotic agents alone and combined with IBP, ASA, and DXP for both clinical isolates of *S. aureus* and *P. aeruginosa* are presented in Table 2.

IBP at 200 µg/mL showed synergistic effects in combination with some antibiotic agents and among MRSA isolates, causing the MIC and MBIC levels to be reduced 4–8-fold for CIP, 2–16-fold for GEN, and 8–32-fold for IPM, MEM, and FEP. In MRSA isolates in the presence of ASA (200 µg/mL), we observed synergistic effects, and the MIC and MBIC were decreased 0–2-fold for CIP and GEN, 2–16-fold for IPM, 2–4-fold for MEM, and 2–8-fold for FEP. In contrast to the synergistic effects of IBP and ASA, in the presence of DXP among MRSA isolates, we observed antagonistic effects, and DXP increased MIC and MBIC levels 2–8-fold for CIP and FEP, 4–16-fold for GEN, and 0–2-fold for IPM, although they had no effect on the levels of MIC and MBIC for MEM. The level of MBEC among MRSA isolates was decreased 2-fold for CIP, GEN, and MEM, 2–4-fold for IPM, and 2–8-fold for FEP in the presence of IBP. MBEC levels in the presence of ASA were reduced 0–2-fold for CIP, GEN, IPM, and FEP among clinical isolates of *S. aureus*. ASA had no effect on the levels of MBEC for MEM among MRSA isolates. IBP at 200 µg/mL in combination with antibiotic agents showed synergistic effects among carbapenem-resistant *P. aeruginosa* and caused the MIC and MBIC levels to be reduced 2–4-fold for CIP, MEM, and FEP, 2-fold for GEN, and 2–8-fold for IPM. Moreover, we observed the synergistic effects and changed levels of MIC and MBIC for some antibiotic agents plus ASA among carbapenem-resistant *P. aeruginosa* isolates. In the presence of ASA at 200 µg/mL level, the MIC and MBIC decreased 0–4-fold for IPM, 2–4-fold for MEM, and 2–8-fold for FEP, and ASA had no effect on the levels of MIC and MBIC for CIP and GEN among carbapenem-resistant *P. aeruginosa* isolates. There were similar findings for MRSA isolates. DXP at 500 µg/mL increased MIC and MBIC 8–16-fold for CIP, 2–8-fold for GEN, 2-fold for IPM and MEM, and 2–4-fold for FEP in carbapenem-resistant *P. aeruginosa* isolates. The level of MBEC of the antibiotic agents was decreased 0–2-fold for CIP, IPM, and FEP in combination with IBP in carbapenem-resistant *P. aeruginosa* isolates, and IBP had no effect on the levels of MBEC for GEN and MEM. Moreover, ASA only reduced 2-fold the level of MBEC for FEP and could not decrease the levels of MBEC for CIP, GEN, IPM, and MEM among carbapenem-resistant *P. aeruginosa* isolates. MBEC levels in the presence of DXP were increased 4-fold for CIP and 0–2-fold for GEN and MEM, and we did not observe any change in MBEC level for IPM and FEP in the presence of DXP among carbapenem-resistant *P. aeruginosa* isolates. The MIC, MBIC, and MBEC level changes among MRSA and carbapenem-resistant *P. aeruginosa* isolates are presented in Table 3 and Table 4, respectively. The MIC, MBIC, and MBEC levels of CIP, GEN, IPM, and MEM among clinical isolates of *S. aureus* and *P. aeruginosa* in combination with IBP, ASA, and DXP are presented in Tables S1–S5 in the supplementary data. Our findings showed that IBP had a greater effect on reducing the MIC and MBIC levels of CIP, GEN, IPM, MEM, and FEP than ASA on both clinical isolates (Table 3 and Table 4). Moreover, IBP had an increased effect on decreasing the levels of MBIC of CIP, GEN, MEM, and FEP compared to the MIC levels in MRSA isolates and an increased effect on reducing the MBIC levels of IMP and CIP compared to MIC among *P. aeruginosa* isolates (Table 3 and Table 4).

**Figure 1:**
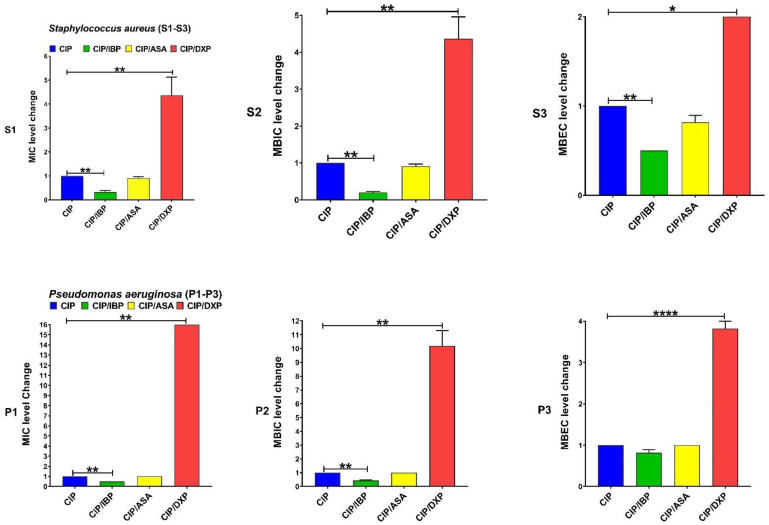
Effects of sub-MICs of ibuprofen (IBP: 200 µg/mL), aspirin (ASA: 200 µg/mL), and dexamethasone sodium phosphate (DXP: 500 µg/mL) on the levels of MIC, MBIC, and MBEC of ciprofloxacin (CIP) for clinical isolates. Graphs were drawn based on fold changes of MIC, MBIC, and MBEC of CIP in combination with sub-MICs of IBP, ASA, and DXP on clinical isolates. Data are displayed as the mean ± standard error of the mean from 3 replicate experiments and were analyzed using ANOVA and nonparametric Kruskal–Wallis test. See S1–S3 for *S. aureus* isolates and P1–P3 for *P. aeruginosa* isolates. IBP significantly decreased the level of MIC and MBIC for CIP among both clinical isolates and MBEC among MRSA. ASA had no significant effects on the level of MIC, MBIC, and MBEC for CIP, and DXP significantly increased the level of MIC, MBIC, and MBEC for CIP among both clinical isolates. *, statistical significance with *p* ≤ 0.05; **, statistical significance with *p* ≤ 0.01; and ****, statistical significance with *p* ≤ 0.0001.

**Figure 2:**
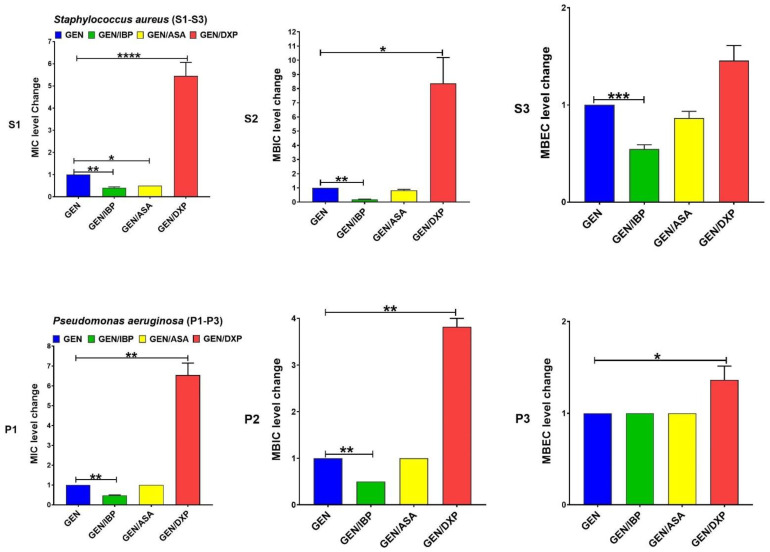
Effects of sub-MICs of ibuprofen (IBP: 200 µg/mL), aspirin (ASA: 200 µg/mL), and dexamethasone sodium phosphate (DXP: 500 µg/mL) on the level of MIC, MBIC, and MBEC of gentamicin (GEN) for clinical isolates. Graphs were drawn based on fold changes of MIC, MBIC, and MBEC for GEN in combination with sub-MICs of IBP, ASA, and DXP on clinical isolates. Data are displayed as the mean ± standard error of the mean from 3 replicate experiments and were analyzed using ANOVA and nonparametric Kruskal–Wallis test. See S1–S3 for *S. aureus* and P1–P3 for *P. aeruginosa*. IBP significantly decreased the levels of MIC, MBIC, and MBEC for GEN among clinical isolate *S. aureus* and MIC and MBIC among *P. aeruginosa* isolates. ASA only significantly decreased MIC for GEN among clinical isolate *S. aureus*. DXP significantly increased the level of MIC and MBIC for GEN among both clinical isolates and MBEC among *P. aeruginosa* isolates. *, statistical significance with *p* ≤ 0.05; **, statistical significance with *p* ≤ 0.01; ***, statistical significance with *p* ≤ 0.001; and ****, statistical significance with *p* ≤ 0.0001.

**Figure 3:**
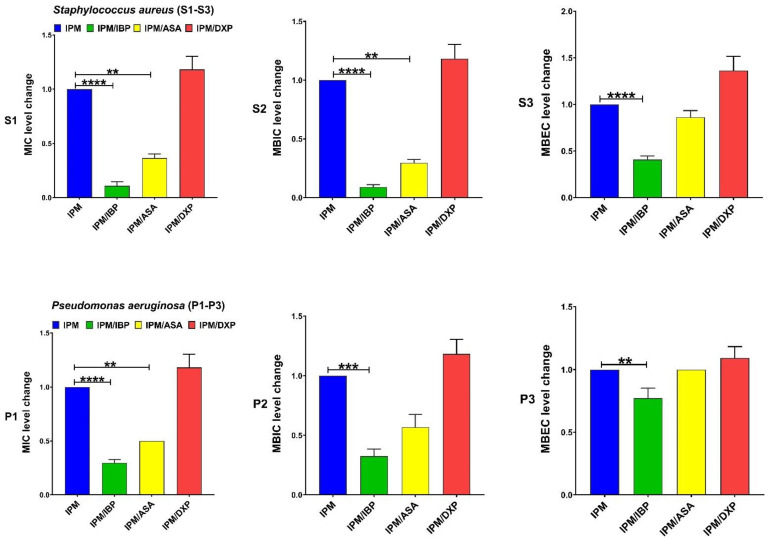
Effects sub-MICs of ibuprofen (IBP: 200 µg/mL), aspirin (ASA: 200 µg/mL), and dexamethasone sodium phosphate (DXP: 500 µg/mL) on the level of MIC, MBIC, and MBEC of imipenem (IPM) for clinical isolates. Graphs were drawn based on fold changes of MIC, MBIC, and MBEC for IPM in combination with sub-MICs of IBP, ASA, and DXP on clinical isolates. Data are displayed as the mean ± standard error of the mean from 3 replicate experiments and were analyzed using ANOVA and nonparametric Kruskal–Wallis test (S1–S3 for *S. aureus* isolates and P1–P3 for *P. aeruginosa* isolates). IBP significantly decreased the levels of MIC, MBIC, and MBEC for IPM among both clinical isolates of *S. aureus* and *P. aeruginosa.* ASA significantly decreased the level of MIC for IPM among both clinical isolates and MBIC for IPM among *S. aureus* isolates. DXP did not significantly affect the MIC, MBC, and MBEC for IPM among both clinical isolates. **, statistical significance with *p* ≤ 0.01; ***, statistical significance with *p* ≤ 0.001; and ****, statistical significance with *p* ≤ 0.0001.

**Figure 4:**
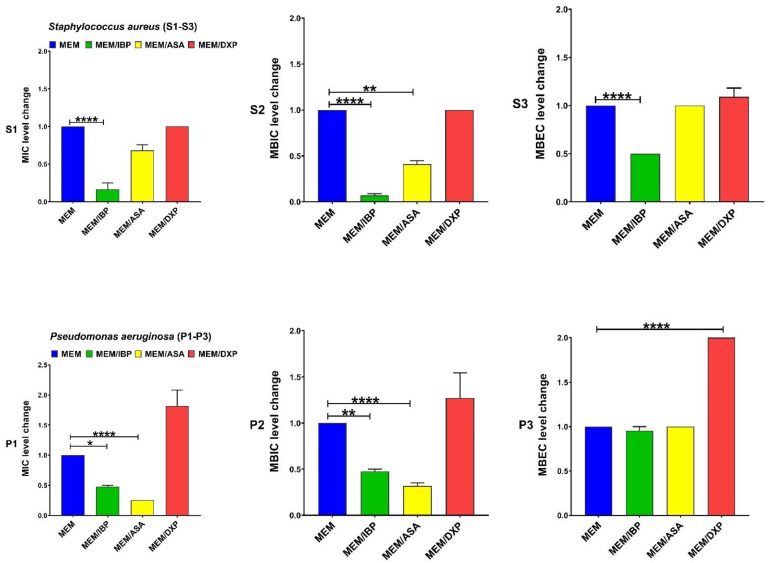
Effects of sub-MICs of ibuprofen (IBP: 200 µg/mL), aspirin (ASA: 200 µg/mL), and dexamethasone sodium phosphate (DXP: 500 µg/mL) on the level of MIC, MBIC, and MBEC of meropenem (MEM) for clinical isolates. Graphs were drawn based on fold changes of MIC, MBIC, and MBEC for MEM in combination with sub-MICs of IBP, ASA, and DXP on clinical isolates. Data are displayed as the mean ± standard error of the mean from 3 replicate experiments and were analyzed using ANOVA and nonparametric Kruskal–Wallis test (S1–S3 for *S. aureus* isolates and P1–P3 for *P. aeruginosa* isolates). IBP significantly decreased the levels of MIC, MBIC, and MBEC for MEM among clinical isolates of *S. aureus* and MIC and MBIC among *P. aeruginosa* isolates. ASA significantly decreased the level of MIC for MEM among clinical isolate *S. aureus* and MIC and MBIC for MEM among *P. aeruginosa* isolates. DXP only significantly increased the level of MBEC for MEM among clinical isolates of *P. aeruginosa*. *, statistical significance with *p* ≤ 0.05; **, statistical significance with *p* ≤ 0.01; and ****, statistical significance with *p* ≤ 0.0001.

**Figure 5:**
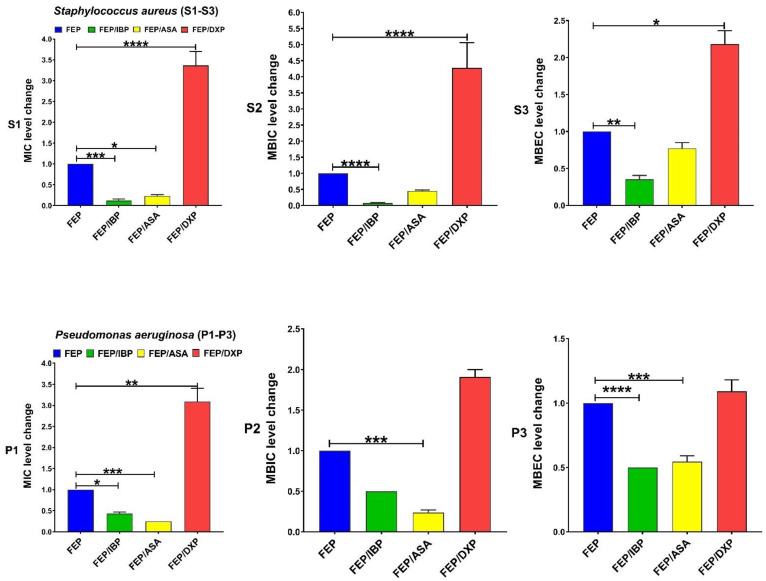
Effects of sub-MICs of Ibuprofen (IBP: 200 µg/mL), aspirin (ASA: 200 µg/mL), and dexamethasone sodium phosphate (DXP: 500 µg/mL) on the levels of MIC, MBIC, and MBEC of cefepime (FEP) for clinical isolates. Graphs were drawn based on fold changes of MIC, MBIC, and MBEC for FEP in combination with sub-MICs of IBP, ASA, and DXP on clinical isolates. Data are displayed as the mean ± standard error of the mean from 3 replicate experiments and were analyzed using ANOVA and nonparametric Kruskal–Wallis test. See S1–S3 for *S. aureus* isolates and P1–P3 for *P. aeruginosa* isolates. IBP significantly decreased the levels of MIC, MBIC, and MBEC for FEP among clinical isolates of *S. aureus* and MIC and MBEC among *P. aeruginosa* isolates. ASA significantly decreased MIC of FEP among clinical isolate *S. aureus* and MIC and MBIC of FEP among *P. aeruginosa*. DXP significantly increased the MIC, MBIC, and MBEC of FEP among clinical isolates of *S. aureus* and MIC among clinical isolates of *P. aeruginosa*. *, statistical significance with *p* ≤ 0.05; **, statistical significance with *p* ≤ 0.01; ***, statistical significance with *p* ≤ 0.001; and ****, statistical significance with *p* ≤ 0.0001.

IBP significantly decreased the levels of MIC and MBIC for CIP among both clinical isolate and MBEC among *S. aureus*. DXP significantly increased the MIC, MBIC, and MBEC for CIP among both clinical isolates, and ASA had no significant effects on MIC, MBIC, and MBEC for CIP (Figure 1). IBP significantly decreased the level of MIC, MBIC, and MBEC for GEN among clinical isolates of *S. aureus* and MIC and MBIC among *P. aeruginosa* isolates. ASA only significantly decreased the level of MIC for GEN among clinical isolates of *S. aureus*. DXP significantly increased the level of MIC and MBIC for GEN among both clinical isolates and MBEC among *P. aeruginosa* isolates (Figure 2). IBP significantly decreased the level of MIC, MBIC, and MBEC for IPM among both clinical isolates of *S. aureus* and *P. aeruginosa.* ASA significantly reduced the level of MIC for IPM among both clinical isolates and MBIC for IPM among *S. aureus* isolates. DXP did not significantly affect the MIC, MBC, and MBEC for IPM among both clinical isolates (Figure 3). IBP significantly decreased the level of MIC, MBIC, and MBEC for MEM among clinical isolates of *S. aureus* and MIC and MBIC among *P. aeruginosa* isolates. ASA significantly decreased the level of MIC for MEM among clinical isolates of *S. aureus* and MIC, and MBIC for MEM among *P. aeruginosa* isolates. DXP only significantly increased the level of MBEC for MEM among clinical isolates of *P. aeruginosa* (Figure 4). IBP significantly decreased the level of MIC, MBIC, and MBEC for FEP among clinical isolates of *S. aureus* and MIC and MBEC among *P. aeruginosa* isolates. ASA significantly decreased the level of MIC for FEP among clinical isolate *S. aureus* and MIC and MBIC for FEP among *P. aeruginosa*. DXP significantly increased the levels of MIC, MBIC, and MBEC for FEP among clinical isolates of *S. aureus* and MIC among clinical isolates of *P. aeruginosa* (Figure 5).

**Figure 6:**
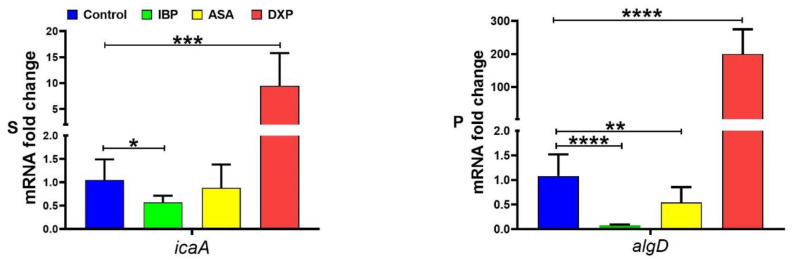
S: The transcriptional level of *icaA* in *S. aureus* with and without the sub-MIC levels of IBP (200 µg/mL), ASA (200 µg/mL), and DXP (500 µg/mL). P: The transcriptional level of *algD* in *P. aeruginosa* with and without the sub-MIC levels of IBP (200 µg/mL), ASA (200 µg/mL), and DXP (500 µg/mL). Graphs were drawn based on fold changes in transcriptional levels of *icaA* and *algD* in clinical isolates of *S. aureus* and *P. aeruginosa*, respectively. Data are displayed as the mean ± standard error of the mean from 3 replicate experiments and were analyzed using the ANOVA test. *, statistical significance with *p* ≤ 0.05; **, statistical significance with *p* ≤ 0.01; ***, statistical significance with *p* ≤ 0.001; and ****, statistical significance with *p* ≤ 0.0001.

Analysis of qPCR experiment results showed changes in the transcriptional levels of *icaA* and *algD* in the presence of 200 µg/mL IBP and ASA and 500 µg/mL DXP compared to the control group. The expression level of *icaA* significantly decreased in the presence of IBP compared to the control. Although the transcriptional level of *icaA* was decreased in the presence of ASA, it was not significant. The expression level of *icaA* in clinical isolates of *S. aureus* in the presence of DXP significantly increased compared to the control (Figure 6). Transcriptional levels of *algD* in *P. aeruginosa* isolates in the presence of IBP and ASA were significantly decreased compared to the control group, and expression levels of *algD* significantly increased in the presence of DXP (Figure 6).

## 4. Discussion

The resistance of bacteria to antibiotic agents is increasing worldwide. The spread of multi-drug resistant (MDR) bacteria such as MRSA or carbapenem-resistant *P. aeruginosa* isolates is a global threat [25]. Anti-inflammatory drugs are commonly used in combination with antibiotics to control the systemic effects of infection [2]. Therefore, evaluating therapeutic formulations that combine the effects of these drugs and antibiotic agents on bacteria can be important. Most studies on anti-inflammatory drugs’ antimicrobial and antibiofilm activity have focused on nonclinical bacterial isolates [11]. In this study, we evaluated the effect of anti-inflammatory drugs, including IBP, ASA, and DXP, on the antibacterial and antibiofilm activity of CIP, GEN, IMP, MEM, and FEP on nonduplicate clinical isolates of carbapenem-resistant *P. aeruginosa* (n = 10) and MRSA (n = 10). We also determined the transcriptional levels of biofilm-related genes, including *icaA* and *algD* in sub-MICs of IBP (200 µg/mL), ASA (200 µg/mL), and DXP (500 µg/mL) in clinical isolates of MRSA and carbapenem-resistant *P. aeruginosa*, respectively. There are different anti-inflammatory drugs, including nonsteroidal, such as aspirin, ibuprofen, diclofenac, and naproxen, and corticosteroidal anti-inflammatory drugs, such as dexamethasone, betamethasone, and hydrocortisone, that are commonly used to ameliorate fever and other symptoms of acute and chronic infections [3]. We describe here that IBP and ASA, combined with some common antibiotics including CIP, GEN, IMP, MEM, and FEP, had decreasing effects on MIC, MBIC, and MBEC. DXP had increasing effects on the MIC, MBIC, and MBEC of some of these antibiotic agents (Table 2, Table 3 and Table 4, Figure 1, Figure 2, Figure 3, Figure 4 and Figure 5).

According to the reports, using a combination of anti-inflammatory drugs and antibiotics to treat infections can have different effects on the MIC, MBIC, and MBEC of antibiotic agents [2,11]. Corticosteroid drugs such as dexamethasone and betamethasone are synthetic analogs of glucocorticoids that have anti-inflammatory and immunosuppressive functions [3]. Many reports show that steroid hormones increase the expression level of virulence and biofilm-associated genes, efflux pump genes associated with MDR, and the rate of replication of bacteria [26,27]. The virulent mucoid biofilm phenotype in *P. aeruginosa* increased in the presence of estradiol [27]. Moreover, estradiol can down-regulate genes involved in nucleotide metabolism and fatty acid biosynthesis and may be associated with enhanced survival and persistence in *Chlamydia trachomatis* [27]. In a study in 2014, it was shown that estradiol compounds increase the planktonic growth and the ability of *Fusobacterium nucleatum* to co-aggregate, and, in addition, it was also shown that polysaccharide production and biofilm formation in *Prevotella intermedia* was enhanced by estradiol in vitro [26]. The effects of using some antimicrobial agents in combination with corticosteroid drugs in vivo have been described. A study in 1996 showed that cloxacillin combined with DXP was more effective than cloxacillin alone in treating bacterial arthritis caused by *S. aureus* in Swiss mice [28]. DXP did not interfere with fluconazole in a murine model of cryptococcosis [29]. Other studies show that the combined use of hydrocortisone with mupirocin and methylprednisolone with imipenem is more effective than the antibiotics alone against eczema and atopic dermatitis by *S. aureus* and severe pneumonia in children, respectively [30,31]. However, some reports have shown the adverse effects on the antimicrobial and antibiofilm activity of antibiotics with corticosteroid drugs such as DXP. A study in 2017 reported that the corticosteroid anti-inflammatory drug DXP abrogates the activity of antimicrobial drugs, including gentamicin, chloramphenicol, oxacillin, ceftriaxone, and meropenem when combined in vitro against planktonic and microbial biofilms of *S. aureus* and *P. aeruginosa* [2]. Studies showed that combining DXP with ceftriaxone and vancomycin in a rabbit model of pneumococcal meningitis causes treatment failure [32,33]. These different effects of using DXP in combination with antibiotics may be due to the antibiotics’ pharmacological properties, the type of bacteria (clinical or nonclinical isolates), and the possibility of DXP interference with the bacterial physiological processes. However, dexamethasone derivatives such as nitro-dexamethasone have recently been introduced, showing acceptable antimicrobial effects. These effects of nitro-dexamethasone have been attributed to the NO group, which can cause antibiofilm activity [34]. Moreover, it has been reported that topical steroids, including fluticasone, mometasone, and budesonide, directly reduce biofilm production and MBIC in vitro in *S. aureus* ATCC 25923 [35]. In this study, as in some other studies, DXP reduced the susceptibility of the isolates to CIP, GEN, and FEP. Interestingly, DXP had no significant effect on the levels of MIC and MBIC of IPM and MEM in both clinical isolates of *S. aureus* and *P. aeruginosa*. Therefore, our findings contrast with the report in 2017 that showed DXP could abrogate MEM activity against clinical isolates of *S. aureus* and *P. aeruginosa* [2]. These findings about IPM and MEM can be considered for their use in combination with DXP, although more studies are needed. However, the differences between our results and other reports may be due to the genotype and clone type of bacteria, antibiotic resistance mechanisms, and growth conditions among bacterial isolates. For example, it has been shown that biofilm production in methicillin-sensitive *S. aureus* isolates (MSSA) is usually associated with polysaccharide intercellular adhesin (PIA) or poly-*N*-acetyl-glucosamine (PNAG), whereas in MRSA isolates the formation of biofilms frequently depends more on the proteinaceous matrix [36,37,38]. Moreover, it has been shown that some clones of MRSA, such as USA300, produce thicker and stronger biofilms than other MRSA clones [38]. The antimicrobial effects of IBP on PAO1 differ from other strains of P. aeruginosa and Gram-negative bacteria such as *Burkholderia* spp [20]. In addition, the pH range and the type of medium are effective on the antimicrobial function of ibuprofen. For example, it has been reported that the MIC levels of IBP against *S. aureus* isolates are 40 to 80 µg/mL at pH 5 [39]. Similarly, it has been observed that growth suppression by IBP on *S. aureus* and *Staphylococcus epidermidis* occurred at concentrations greater than 150 µg/mL to 450 µg/mL at a pH of 7 [40]. However, the type of growth media can have effects on gene expression, metabolic functions, and other physiological growth conditions of bacteria [20].

Similar to other studies, our results show that ASA and IBP, in combination with some antibiotic agents, can reduce the MIC, MBIC, and MBEC. A few reports have shown the antibacterial and antifungal activity of IBP and its synergy with antibiotic agents. In 2017, it was reported that ASA, IBP, and diclofenac have antibacterial activity against Gram-positive and Gram-negative pathogenic bacteria such as MRSA and *P. aeruginosa* [1]. Moreover, a synergism effect between IBP/ASA in combination with cefuroxime and chloramphenicol was reported on *S. aureus* [1].

IBP has been shown to have antifungal activity in vitro against dermatophytes with MIC 5–40 μg/mL [39]. A study has demonstrated that combining IBP with fluconazole resulted in synergic activity against *Candida* spp., and MICs of fluconazole among the fluconazole-resistant strains decreased 2–128-fold in combination with IBP [41]. Recently, it has been shown that the morphogenesis and pathogenicity of fungi can be affected by cyclooxygenase (COX) inhibitors such as ASA, IBP, and indomethacin, combined with antifungal drugs [42]. Inhibitors of cyclooxygenase iso-enzymes such as ASA and diclofenac effectively decrease the germ tube formation of *Candida albicans* isolates [43]. Studies have shown that NSAID compounds can reduce the ability of biofilm formation in *Candida* spp., and the combination of NSAIDs with antifungal drugs can have synergistic effects [41,42]. These effects may be due to the inhibition of prostaglandin E2 (PGE2) due to the inhibition of COX by NSAIDs [42,43]. However, the mechanistic action of NSAIDs on bacteria is not clear.

One of the important points about the antimicrobial and antibiofilm activity of anti-inflammatory drugs is to pay attention to the concentration of their antimicrobial function in vitro compared to their plasma concentration. This concentration in humans for ASA is from 50 to 200 µg/mL, which are doses with antimicrobial and antibiofilm activity against some microorganisms [44]. Moreover, it has been shown that the antiquorum sensing and antibiofilm activity of IBP against *P. aeruginosa* is related to rising drug concentrations [45].

During infection, biofilm formation by bacteria plays an important role in bacterial colonization, antibiotic resistance, and the immune system [10]. The production of exopolysaccharides or extracellular polymeric substances (EPS) is important in biofilm formation in bacteria [10]. In *S. aureus*, the major exopolysaccharide produced for biofilm formation is termed polysaccharide intercellular adhesion (PIA), also known as poly-*N*-acetyl-glucosamine (PNAG) [10]. The enzymes that synthesize the PIA/PNAG are encoded by the *icaADBC* operon [10]. Among the *ica* operon genes, *icaA* and *icaD* encode transmembrane proteins involved in oligomer synthesis for PNAG formation [10]. In this study, DXP increased the transcriptional level of *icaA*, from which it can be concluded that steroid compounds such as DXP increase the production of biofilms by increasing the expression of operon *ica* genes and thus reduce the susceptibility to some antibiotic agents in *S. aureus*. However, in this study ASA as an NSAID drug did not show significant changes in *icaA* expression, although IBP significantly decreased the transcriptional level of the *icaA* in *S. aureus*.

Alginate overproduction by increasing biofilm formation can protect *P. aeruginosa* from phagocytosis and antibiotic penetration [6]. *algD* is the main operon involved in biofilm formation in *P. aeruginosa* and encodes the main enzymes for alginate synthesis [5,6]. In the present study, DXP increased the transcriptional level of *algD*, from which it can be concluded that steroid compounds such as DXP increase the production of alginate or biofilm by increasing the expression of *algD* operon genes and thus reduce the susceptibility of *P. aeruginosa* isolates to antibiotic agents. However, ASA and IBP reduced the expression level of *algD* in the present study, which may explain the decrease in susceptibility of bacteria to various antibiotics in the presence of these drugs. Decreased expression of biofilm-associated genes, including *icaA* and *algD* in the presence of IBP and ASA, may explain the reduction in the MBIC of antibiotics agents among both clinical isolates, and, in some cases, this reduction in the MBIC level was more than the MIC (Table 3 and Table 4). However, some studies have reported that NSAIDs can reduce antibiotic susceptibility and bacterial pathogenicity through different mechanisms. IBP potentially uncouples oxidative phosphorylation in bacteria and causes depletion in the intracellular ATP concentration in *P. aeruginosa* PAO1 [20]. Another study reported that IBP and ASA could bind to DNA gyrase and inhibit the growth of bacteria such as antibiotic agents. Recent chemoinformatics- and bioinformatics-based studies have shown that IBP has a similar structure to the quinolones and fluoroquinolones classes of antimicrobials [46]. In addition to the antibiofilm activity of NSAIDs against *S. aureus*, there is evidence pointing to the activity of NSAIDs against the pathogenicity of this bacterium by antivirulence properties such as the inhibition of hemolysis and staphyloxanthin production in this bacterium [47]. ASA has antibacterial and antibiofilm activity against *S. aureus* by blocking *agrA*-regulated virulence genes and down-regulating the expression of biofilm-associated genes such as *icaA* and *fnbA.* However, in our study, *icaA* expression was down-regulated in the presence of ASA but was not statistically significant [11]. Intercellular signaling, often known as quorum sensing (QS), is involved in biofilm development, and it has been shown that ASA can inhibit quorum sensing in *P. aeruginosa* [11,48]. A study demonstrated that ibuprofen could inhibit biofilm formation and quorum sensing in P. aeruginosa, which is probably related to the binding of ibuprofen to LuxR, LasR, LasI, and RhlR proteins [45].

## 5. Conclusions

This study shows that the antimicrobial and antibiofilm ability of some antibiotic agents increased in the presence of IBP and ASA as NSAIDs. Moreover, DXP reduces the susceptibility of bacteria to ciprofloxacin, gentamicin, and cefepime by increasing biofilm production, but it does not affect the activity of the imipenem and meropenem. This increase or decrease in the susceptibility of bacteria to antibiotic agents in the presence of the anti-inflammatory drug, in addition to their type and concentration, may be related to the type or clone of bacteria, physiological conditions of bacteria, and antibiotics class. With the increasing resistance to antibiotic agents among bacteria, proposing new strategies to treat infections and new antibacterial compounds is important. According to our findings, although more studies are needed, formulation treatments including NSAIDs such as ibuprofen and its derivatives alone or in combination with antibiotic agents can be suggested as antibiofilm compounds for treating bacterial-biofilm-associated infections. However, the data in this work show that dexamethasone, in interaction with antibiotics agents, may have adverse effects on their function, which is suggested to be considered in the treatment of infections. Finally, the results in this work highlight interactions between different antibiotics and common anti-inflammatory drugs and provide essential insights into the design and development of novel compounds or a new formulation strategy for treating bacterial infections.

## Figures and Tables

**Table 2:** Distribution of MIC, MBIC, and MBEC 50 and 90 in clinical isolates of *S. aureus* and *P. aeruginosa* with and without sub-MICs of anti-inflammatory drugs.

Antibiotics Agents	MIC, MBIC, and MBEC (µg/mL) 50 and 90 for Clinical Isolates of *S. aureus* Isolates						MIC, MBIC, and MBEC (µg/mL) 50 and 90 for Clinical Isolates of *P. aeruginosa*					
	MIC_50_	MIC_90_	MBIC_50_	MBIC_90_	MBEC_50_	MBEC_90_	MIC_50_	MIC_90_	MBIC_50_	MBIC_90_	MBEC_50_	MBEC_90_
CIP	64	128	128	256	1024	2048	8	16	32	64	512	512
CIP/IBP	16	32	32	64	1024	2048	4	8	16	16	256	512
CIP/ASA	64	128	128	256	1024	2048	8	16	32	64	512	512
CIP/DXP	256	256	256	1024	4096	≥8192	128	256	256	1024	2048	2048
GEN	64	64	256	256	1024	1024	16	32	64	128	1024	1024
GEN/IBP	32	32	64	64	512	1024	8	16	32	64	1024	1024
GEN/ASA	32	32	128	256	1024	1024	16	32	64	128	1024	1024
GEN/DXP	256	256	1024	1024	1024	2048	128	128	256	512	1024	1024
IPM	16	32	64	64	1024	2048	32	32	512	512	2048	2048
IPM/IBP	1	2	2	8	512	512	8	16	64	256	1024	2048
IPM/ASA	8	8	8	16	1024	2048	16	16	128	256	2048	2048
IPM/DXP	64	64	32	64	2048	2048	32	32	256	512	2048	2048
MEM	16	32	64	128	1024	2048	32	32	64	128	1024	1024
MEM/IBP	1	2	4	8	512	1024	16	16	32	64	1024	1024
MEM/ASA	16	16	16	64	1024	2048	8	8	16	32	1024	1024
MEM/DXP	16	32	64	128	1024	2048	32	64	64	128	1024	2048
FEP	16	64	64	256	2048	2048	32	64	512	512	4096	4096
FEP/IBP	2	4	4	8	512	512	16	32	256	256	2048	2048
FEP/ASA	8	8	32	64	1024	1024	8	16	64	128	2048	2048
FEP/DXP	64	128	256	1024	2048	4096	128	128	512	1024	4096	4096

CIP: ciprofloxacin, GEN: gentamicin, IPM: imipenem, MEM: meropenem, FEP: cefepime, IBP: ibuprofen, ASA: acetylsalicylic acid, DXP: dexamethasone sodium phosphate, MIC: minimum inhibitory concentration, MBIC: minimum biofilm inhibitory concentration, MBEC: minimum biofilm eradication concentration.

**Table 3:** The level changes of the MIC, MBIC, and MBEC of antibiotic agents with/without sub-MICs of anti-inflammatory drugs among MRSA isolates.

Drugs	MIC Range	MIC Fold Changes Range	Isolates n (%)	*p*-Value	MBIC Range	MBIC Fold Changes Range	Isolates n (%)	*p*-Value	MBEC Range	MBEC Fold Changes Range	Isolates n (%)	*p*-Value
CIP	16–128	C	-	-	64–256	C	-	-	1024–4096	C	-	
CIP + IBP	4–32	4 ↓	10 (100)	0.0058	8–64	4–8 ↓	10 (100)	0.0051	512–2048	2 ↓	10 (100)	0.0039
CIP + ASA	16–128	0–2 ↓	2 (20)	>0.9999	128–256	0–2 ↓	2 (20)	>0.9999	1024–2048	0–2 ↓	4 (40)	0.7261
CIP + DXP	128–256	2–8 ↑	10 (100)	0.0055	256–1024	2–8 ↑	10 (100)	0.0058	2048–8192	2 ↑	10 (100)	0.0141
GEN	8–64	C	-	-	64–256	C	-	-	1024–2048	C	-	-
GEN + IBP	2–32	2–4 ↓	10 (100)	0.0012	4–64	4–16 ↓	10 (100)	0.0013	512–1024	2 ↓	10 (100)	0.0007
GEN + ASA	4–32	2 ↓	10 (100)	0.0166	64–128	0–2 ↓	3 (100)	>0.9999	1024	2 ↓	3 (30)	0.8025
GEN + DXP	64–256	4–8 ↑	10 (100)	0.0001	1024	4–16 ↑	10 (100)	0.0176	1024–2048	4 ↑	4 (40)	0.4255
IPM	8–32	C	-	-	32–64	C	-	-	1024–2048	C	-	-
IPM + IBP	0.5–2	8–32 ↓	10 (100)	<0.0001	2–8	8–16 ↓	10 (100)	<0.0001	512	2–4 ↓	10 (100)	<0.0001
IPM + ASA	4–8	2–4 ↓	10 (100)	0.0052	8–16	2–4 ↓	10 (100)	0.0028	512–2048	0–2 ↓	20 (20)	0.9313
IPM + DXP	16–32	2 ↑	2 (20)	>0.9999	32–64	0–2 ↑	2 (20)	>0.9999	1024–2048	0–2 ↑	4 (40)	0.7792
MEM	8–32	C	-	-	32–128	C	-	-	1024–2048	C	-	-
MEM + IBP	1–2	8–16 ↓	10 (100)	<0.0001	1–8	16–32 ↓	10 (100)	<0.0001	512–1024	2 ↓	10 (10)	<0.0001
MEM + ASA	4–16	0–2 ↓	7 (70)	0.0707	16–64	2–4 ↓	10 (100)	0.0032	1024–2048	0	-	>0.9999
MEM + DXP	8–32	0	-	>0.9999	32–128	0	-	>0.9999	1024–2048	0	-	>0.9999
FEP	16–64	C	-	-	32–256	C	-	-	1024–2048	C	-	-
FEP + IBP	1–4	8–16 ↓	10 (100)	0.0003	2–8	16–32 ↓	10 (100)	<0.0001	256–1024	2–8 ↓	10 (100)	0.0023
FEP + ASA	2–8	4–8 ↓	10 (100)	0.0468	16–64	2–4 ↓	10 (100)	0.0934	1024–2048	2 ↓	5 (50)	0.6736
FEP + DXP	64–128	2–4 ↑	10 (100)	<0.0001	64–1024	2–8 ↑	10 (100)	<0.0001	2048–4096	2 ↑	10 (100)	0.0226

CIP: ciprofloxacin, GEN: gentamicin, IPM: imipenem, MEM: meropenem, FEP: cefepime, IBP: ibuprofen, ASA: acetylsalicylic acid, DXP: dexamethasone sodium phosphate, MIC: minimum inhibitory concentration, MBIC: minimum biofilm inhibitory concentration, MBEC: minimum biofilm eradication concentration, C: control, ↑: increase, and ↓: decrease.

**Table 4:** The level changes of the MIC, MBIC, and MBEC of antibiotic agents with/without sub-MICs of anti-inflammatory drugs among carbapenem-resistant *P. aeruginosa*.

Drugs	MIC Range	MIC Fold Changes Range	Isolates n (%)	*p*-Value	MBIC Range	MBIC Fold Changes Range	Isolates n (%)	*p*-Value	MBEC Range	MBEC Fold Changes Range	Isolates n (%)	*p*-Value
CIP	4–16	C	-	-	32–64	C	-	-	256–512	C	-	-
CIP + IBP	2–8	2 ↓	10 (100)	0.0031	16	2–4 ↓	10 (100)	0.0035	256–512	0–2 ↓	4 (40)	0.5728
CIP + ASA	4–16	0	-	>0.9999	32–64	0	0	>0.9999	256–512	0	-	>0.9999
CIP + DXP	64–256	16 ↑	10 (100)	0.0031	256–1024	8–16 ↑	10 (100)	0.0035	1024–2048	4 ↑	10 (100)	<0.0001
GEN	16–32	C	-	-	64–128	C	-	-	512–1024	C	-	-
GEN + IBP	8–16	2 ↓	10 (100)	0.0035	32–64	2 ↓	10 (100)	0.0032	512–1024	0	-	>0.9999
GEN + ASA	16–32	0	-	>0.9999	64–128	0	0	>0.9999	512–1024	0	-	>0.9999
GEN + DXP	128	4–8 ↑	10 (100)	0.0035	256–512	2–4 ↑	10 (100)	0.0032	1024	0–2 ↑	3 (30)	0.0101
IMP	8–32	C	-	-	16–512	C	-	-	1024–2048	C	-	-
IMP + IBP	2–16	2–4 ↓	10 (100)	<0.0001	8–256	2–8 ↓	10 (100)	0.0001	1024–2048	0–2 ↓	4 (40)	0.0082
IMP + ASA	4–16	2 ↓	10 (100)	0.0038	32–256	0–4 ↓	60 (60)	0.00191	1024–2048	0	-	>0.9999
IMP + DXP	16–32	2 ↑	2 (20)	>0.9999	32–512	2 ↑	2 (20)	>0.9999	1024–2048	0	-	>0.9999
MEM	16–32	C	-	-	64–128	C	-	-	1024	C	-	-
MEM + IBP	8–16	2 ↓	10 (100)	0.0313	32–64	2 ↓	10 (100)	0.0012	1024	0	-	>0.9999
MEM + ASA	4–8	4 ↓	10 (100)	<0.0001	32	2–4 ↓	10 (100)	<0.0001	1024	0	-	>0.9999
MEM + DXP	32–64	2 ↑	6 (60)	0.5526	64–128	0	-	>0.9999	1024–2048	2 ↑	10 (100)	<0.0001
FEP	32–64	C	-	-	256–512	C	-	-	4096	C	-	-
FEP + IBP	8–32	2–4 ↓	10 (100)	0.0153	128–256	2 ↓	10 (100)	0.0724	2048	2 ↓	10 (100)	<0.0001
FEP + ASA	8–16	4 ↓	10 (100)	0.0003	64–128	2–8 ↓	10 (100)	0.0001	2048	2 ↓	10 (100)	0.0001
FEP + DXP	128	2–4 ↑	10 (100)	0.0001	512–1024	2 ↑	10 (100)	0.1806	4096	0 ↑	-	>0.9999

CIP: ciprofloxacin, GEN: gentamicin, IPM: imipenem, MEM: meropenem, FEP: cefepime, IBP: ibuprofen, ASA: acetylsalicylic acid, DXP: dexamethasone sodium phosphate, MIC: minimum inhibitory concentration, MBIC: minimum biofilm inhibitory concentration, MBEC: minimum biofilm eradication concentration, C: control, ↑: increase, and ↓: decrease.

## Data Availability

All data is included in the main manuscript and in the Supplementary Materials.

## Supplementary Materials

The following supporting information can be downloaded at: https://www.mdpi.com/article/10.3390/pharmaceutics15010004/s1, Table S1. The MIC, MBIC, and MBEC to ciprofloxacin (µg/mL) alone and with sub-MIC of IBP, ASA, and DXP as anti-inflammatory drugs. Table S2. The MIC, MBIC, and MBEC to gentamicin (µg/mL) alone and with sub-MIC of IBP, ASA, and DXP as anti-inflammatory drugs. Table S3. The MIC, MBIC, and MBEC to imipenem (µg/mL) alone and with sub-MIC of IBP, ASA, and DXP as anti-inflammatory drugs. Table S4. The MIC, MBIC, and MBEC (µg/mL) to meropenem alone and with sub-MIC of IBP, ASA, and DXP as anti-inflammatory drugs. Table S5. The MIC, MBIC, and MBEC (µg/mL) to cefepime alone and with sub-MIC of IBP, ASA, and DXP as anti-inflammatory drugs.
