# Supplementary material for: Antimicrobial and Antibiofilm Effects of Combinatorial Treatment Formulations of Anti-Inflammatory Drugs—Common Antibiotics against Pathogenic Bacteria
Source: Pharmaceutics. 2022 Dec 20;15(1):4. doi: 10.3390/pharmaceutics15010004 (PMC9864814; doi:10.3390/pharmaceutics15010004)
Supplement: Supplementary file 1 [file pharmaceutics-15-00004-s001.zip › pharmaceutics-2022855-supplementary.pdf]

**Supplemental results Tables S1-S5.**

**RP62A:** *Staphylococcus epidermidis* RP62A (ATCC 35984).

**PAO1:** *Pseudomonas aeruginosa* PAO1.

**Table S1. The MIC, MBIC, and MBEC to ciprofloxacin (µg/mL) alone and with sub-MIC of IBP, ASA, and DXP as anti-inflammatory drugs.**

| **Antibacterial/ anti-inflammatory drugs** | **MIC/MBIC/MBEC (µg/mL)** | **Standard strain** | | **Clinical isolates** | | | | | | |
|---|---|---|---|---|---|---|---|---|---|---|
| | | **PAO1** | **RP62A** | ***S. aureus*** (n=4) | ***S. aureus*** (n=2) | ***S. aureus*** (n=2) | ***S. aureus*** (n=2) | ***P. aeruginosa*** (n=4) | ***P. aeruginosa*** (n=3) | ***P. aeruginosa*** (n=3) |
| **IBP** | **MIC** | **2048** | **1024** | **1024** | **1024** | **1024** | **1024** | **1024** | **1024** | **2048** |
| **ASA** | | **8192** | **2048** | **8192** | **4096** | **4096** | **4096** | **4096** | **4096** | **8192** |
| **DXP** | | - | - | - | - | - | - | - | - | - |
| **CIP** | | **0.125** | **1** | **128** | **64** | **32** | **16** | **4** | **16** | **8** |
| CIP+IBP | | ≤0.03125 | 0.125 | 32 | 16 | 8 | 4 | 2 | 8 | 4 |
| CIP+ASA | | 0.125 | 0.5 | 128 | 64 | 16 | 16 | 4 | 16 | 8 |
| CIP+DXP | | 2 | 4 | 256 | 256 | 128 | 128 | 64 | 256 | 128 |
| **CIP** | **MBIC** | **8** | **16** | **256** | **128** | **128** | **64** | **32** | **64** | **32** |
| IBP | | 4096 | 4096 | 4096 | 4096 | 4096 | 4096 | 4096 | 4096 | 8192 |
| ASA | | - | - | - | - | - | - | - | - | - |
| DXP | | - | - | - | - | - | - | - | - | - |
| CIP+IBP | | 4 | 4 | 64 | 32 | 16 | 8 | 16 | 16 | 16 |
| CIP+ASA | | 8 | 16 | 256 | 64 | 128 | 64 | 32 | 64 | 32 |
| CIP+DXP | | 64 | 64 | 1024 | 1024 | 256 | 256 | 256 | 1024 | 256 |
| **CIP** | **MBEC** | **512** | **512** | **4096** | **2048** | **1024** | **1024** | **256** | **512** | **512** |
| IBP | | - | - | - | - | - | - | - | - | - |
| ASA | | - | - | - | - | - | - | - | - | - |
| DXP | | - | - | - | - | - | - | - | - | - |
| CIP+IBP | | 256 | 512 | 2048 | 1024 | 512 | 512 | 256 | 256 | 512 |
| CIP+ASA | | 512 | 512 | 2048 | 2048 | 1024 | 1024 | 256 | 512 | 512 |
| CIP+DXP | | 1024 | 1024 | ≥8192 | 4096 | 2048 | 2048 | 1024 | 2048 | 2048 |

**MIC:** Minimum Inhibitory Concentration, **MBIC:** Minimal Biofilm Inhibitory Concentration, **MBEC:** Minimal Biofilm Eradication Concentration, **CIP:** Ciprofloxacin, **DXP:** Dexamethasone sodium phosphate, **ASA:** Aspirin (Acetylsalicylic acid), **IBP:** Ibuprofen. - : No antibacterial or anti-biofilm effects were observed.

**Table S2. The MIC, MBIC, and MBEC to gentamicin (µg/mL) alone and with sub-MIC of IBP, ASA, and DXP as anti-inflammatory drugs.**

| Antibacterial/ anti-inflammatory drugs | MIC/MBIC/MBEC (µg/mL) | Standard strains | | Clinical isolates | | | | | | |
|---|---|---|---|---|---|---|---|---|---|---|
| | | PAO1 | RP62A | *S. aureus* (n=3) | *S. aureus* (n=3) | *S. aureus* n=2) | *S. aureus* (n=2) | *P. aeruginosa* (n=3) | *P. aeruginosa* (n=3) | *P. aeruginosa* (n=4) |
| **IBP** | **MIC** | **2048** | **1024** | **1024** | **1024** | **1024** | **1024** | **1024** | **1024** | **2048** |
| **ASA** | | **8192** | **2048** | **4096** | **4096** | **8192** | **8192** | **4096** | **4096** | **8192** |
| **DXP** | | - | - | - | - | - | - | - | - | - |
| **GEN** | | **1** | **64** | **64** | **64** | **16** | **8** | **16** | **16** | **32** |
| GEN+IBP | | 0.25 | 32 | 32 | 32 | 4 | 2 | 8 | 8 | 16 |
| GEN+ASA | | 1 | 32 | 32 | 32 | 8 | 4 | 16 | 16 | 32 |
| GEN+DXP | | 8 | 256 | 256 | 256 | 128 | 64 | 128 | 128 | 128 |
| **GEN** | **MBIC** | **8** | **256** | **256** | **256** | **64** | **64** | **64** | **64** | **128** |
| IBP | | **4096** | **2048** | **4096** | **4096** | **2048** | **2048** | **4096** | **2048** | **4096** |
| ASA | | - | - | - | - | - | - | - | - | - |
| DXP | | - | - | - | - | - | - | - | - | - |
| GEN+IBP | | 4 | 32 | 64 | 64 | 8 | 4 | 32 | 32 | 64 |
| GEN+ASA | | 8 | 128 | 128 | 256 | 64 | 64 | 64 | 64 | 128 |
| GEN+DXP | | 32 | 1024 | 1024 | 1024 | 1024 | 1024 | 256 | 256 | 512 |
| **GEN** | **MBEC** | **512** | **1024** | **2048** | **1024** | **1024** | **1024** | **512** | **1024** | **1024** |
| IBP | | - | - | - | - | - | - | - | - | - |
| ASA | | - | - | - | - | - | - | - | - | - |
| DXP | | - | - | - | - | - | - | - | - | - |
| GEN+IBP | | 512 | 512 | 1024 | 512 | 512 | 512 | 512 | 1024 | 1024 |
| GEN+ASA | | 512 | 1024 | 1024 | 1024 | 1024 | 1024 | 512 | 1024 | 1024 |
| GEN+DXP | | 1024 | 1024 | 2048 | 1024 | 2048 | 2048 | 1024 | 1024 | 1024 |

**MIC:** Minimum Inhibitory Concentration**, MBIC:** Minimal Biofilm Inhibitory Concentration**, MBEC:** Minimal Biofilm Eradication Concentration**, GEN:** Gentamicin**, DXP:** Dexamethasone sodium phosphate**, ASA:** Aspirin (Acetylsalicylic acid)**, IBP:** Ibuprofen. **-** **:** No antibacterial or anti-biofilm effects were observed.

**Table S3. The MIC, MBIC, and MBEC to imipenem (µg/mL) alone and with sub-MIC of IBP, ASA, and DXP as anti-inflammatory drugs.**

| Antibacterial/anti-inflammatory drugs | MIC/MBIC/MBEC (µg/mL) | Standard strains | | Clinical isolates | | | | | | | |
|---|---|---|---|---|---|---|---|---|---|---|---|
| | | PAO1 | RP62A | *S. aureus* (n=4) | *S. aureus* (n=2) | *S. aureus* (n=2) | *S. aureus* (n=2) | *P. aeruginosa* (n=4) | *P. aeruginosa* (n=2) | *P. aeruginosa* (n=2) | *P. aeruginosa* (n=2) |
| **IBP** | **MIC** | **2048** | **1024** | **1024** | **1024** | **1024** | **1024** | **1024** | **2048** | **1024** | **1024** |
| **ASA** | | **8192** | **2048** | **8192** | **4096** | **4096** | **4096** | **4096** | **8192** | **4096** | **4096** |
| **DXP** | | - | - | - | - | - | - | - | - | - | - |
| **IPM** | | **0.5** | **0.25** | **32** | **16** | **16** | **8** | **32** | **32** | **16** | **8** |
| IPM+IBP | | 0.125 | 0.125 | 2 | 0.5 | 1 | 1 | 8 | 16 | 4 | 2 |
| IPM+ASA | | 0.25 | 0.125 | 8 | 4 | 8 | 4 | 16 | 16 | 8 | 4 |
| IPM+DXP | | 0.5 | 0.25 | 32 | 16 | 16 | 16 | 32 | 32 | 16 | 16 |
| **IPM** | **MBIC** | **2** | **1** | **64** | **32** | **64** | **32** | **512** | **512** | **32** | **16** |
| IBP | | 4096 | 2048 | 4096 | 4096 | 4096 | 4096 | 4096 | 8192 | 4096 | 4096 |
| ASA | | - | - | - | - | - | - | - | - | - | - |
| DXP | | - | - | - | - | - | - | - | - | - | - |
| IPM+IBP | | 0.125 | 0.25 | 2 | 2 | 8 | 4 | 64 | 256 | 16 | 8 |
| IPM+ASA | | 0.5 | 0.25 | 16 | 8 | 16 | 16 | 128 | 256 | 32 | 16 |
| IPM+DXP | | 2 | 1 | 32 | 32 | 64 | 64 | 256 | 512 | 32 | 32 |
| **IPM** | **MBEC** | **512** | **128** | **2048** | **1024** | **1024** | **1024** | **2048** | **2048** | **1024** | **1024** |
| IBP | | - | - | - | - | - | - | - | - | - | - |
| ASA | | - | - | - | - | - | - | - | - | - | - |
| DXP | | - | - | - | - | - | - | - | - | - | - |
| IPM+IBP | | 256 | 64 | 512 | 512 | 512 | 512 | 1024 | 2048 | 1024 | 1024 |
| IPM+ASA | | 512 | 64 | 2048 | 512 | 1024 | 1024 | 2048 | 2048 | 1024 | 1024 |
| IPM+DXP | | 1024 | 128 | 2048 | 1024 | 2048 | 2048 | 2048 | 2048 | 1024 | 1024 |

**MIC:** Minimum Inhibitory Concentration, **MBIC:** Minimal Biofilm Inhibitory Concentration, **MBEC:** Minimal Biofilm Eradication Concentration, **IPM:** Imipenem, **DXP:** Dexamethasone sodium phosphate, **ASA:** Aspirin (Acetylsalicylic acid), **IBP:** Ibuprofen. - : No antibacterial or anti-biofilm effects were observed.

**Table S4. The MIC, MBIC, and MBEC (µg/mL) to meropenem alone and with sub-MIC of IBP, ASA, and DXP as anti-inflammatory drugs.**

| Antibacterial/anti-inflammatory drugs | MIC/MBIC/MBEC (µg/mL) | Standard strains | | Clinical isolates | | | | | |
|---|---|---|---|---|---|---|---|---|---|
| | | PAO1 | RP62A | *S. aureus* (n=4) | *S. aureus* (n=3) | *S. aureus* (n=3) | *P. aeruginosa* (n=3) | *P. aeruginosa* (n=4) | *P. aeruginosa* (n=3) |
| **IBP** | **MIC** | **1024** | **1024** | **1024** | **1024** | **1024** | **1024** | **2048** | **1024** |
| **ASA** | | **8192** | **2048** | **8192** | **4096** | **4096** | **4096** | **8192** | **4096** |
| **DXP** | | - | - | - | - | - | - | - | - |
| **MEM** | | **0.5** | **≤0.125** | **32** | **16** | **8** | **32** | **32** | **16** |
| MEM+IBP | | 0.125 | 0.125 | 2 | 1 | 1 | 16 | 16 | 8 |
| MEM+ASA | | 0.125 | 0.125 | 16 | 16 | 4 | 8 | 8 | 4 |
| MEM+DXP | | 2 | 0.125 | 32 | 16 | 8 | 64 | 32 | 32 |
| **MEM** | **MBIC** | **2** | **0.5** | **128** | **64** | **32** | **64** | **128** | **64** |
| IBP | | 4096 | 2048 | 4096 | 4096 | 4096 | 4096 | 8192 | 4096 |
| ASA | | - | - | - | - | - | - | - | - |
| DXP | | - | - | - | - | - | - | - | - |
| MEM+IBP | | 0.5 | 0.125 | 8 | 4 | 1 | 32 | 64 | 32 |
| MEM+ASA | | 0.5 | 0.125 | 64 | 16 | 16 | 16 | 32 | 32 |
| MEM+DXP | | 8 | 0.5 | 128 | 64 | 32 | 64 | 128 | 64 |
| **MEM** | **MBEC** | **512** | **512** | **2048** | **1024** | **1024** | **1024** | **1024** | **1024** |
| IBP | | - | - | - | - | - | - | - | - |
| ASA | | - | - | - | - | - | - | - | - |
| DXP | | - | - | - | - | - | - | - | - |
| MEM+IBP | | 256 | 256 | 1024 | 512 | 512 | 1024 | 1024 | 1024 |
| MEM+ASA | | 512 | 512 | 2048 | 1024 | 1024 | 1024 | 1024 | 1024 |
| MEM+DXP | | 1024 | 1024 | 2048 | 1024 | 1024 | 2048 | 2048 | 1024 |

**MIC:** Minimum Inhibitory Concentration**, MBIC:** Minimal Biofilm Inhibitory Concentration**, MBEC:** Minimal Biofilm Eradication Concentration**, MEM:** Meropenem**, DXP:** Dexamethasone sodium phosphate**, ASA:** Aspirin (Acetylsalicylic acid), **IBP:** Ibuprofen. **-** **:** No antibacterial or anti-biofilm effects were observed.

| Table S5. The MIC, MBIC, and MBEC (µg/mL) to cefepime alone and with sub-MIC of IBP, ASA, and DXP as anti-inflammatory drugs. | | | | | | | | | | |
|---|---|---|---|---|---|---|---|---|---|---|
| Antibacterial/anti-inflammatory drugs | MIC/MBIC/MBEC (µg/mL) | Standard strains | | Clinical isolates | | | | | | |
| | | PAO1 | RP62A | *S. aureus* (n=2) | *S. aureus* (n=3) | *S. aureus* (n=3) | *S. aureus* (n=2) | *P. aeruginosa* (n=4) | *P. aeruginosa* (n=3) | *P. aeruginosa* (n=3) |
| **IBP** | **MIC** | **2048** | **1024** | **1024** | **1024** | **1024** | **1024** | **2048** | **1024** | **1024** |
| ASA | | 8192 | 4096 | 8192 | 8192 | 4096 | 4096 | 8192 | 4096 | 4096 |
| DXP | | - | - | - | - | - | - | - | - | - |
| FEP | | 1 | 0.5 | 64 | 32 | 16 | 16 | 64 | 32 | 32 |
| FEP+IBP | | 0.5 | 0.25 | 4 | 2 | 2 | 1 | 32 | 16 | 8 |
| FEP+ASA | | 0.25 | 0.25 | 8 | 8 | 4 | 2 | 16 | 8 | 8 |
| FEP+DXP | | 2 | 0.25 | 128 | 128 | 64 | 64 | 128 | 128 | 128 |
| **FEP** | **MBIC** | **4** | **0.5** | **256** | **128** | **32** | **64** | **512** | **256** | **512** |
| IBP | | 4096 | 4096 | 4096 | 4096 | 4096 | 4096 | 8192 | 4096 | 4096 |
| ASA | | **-** | **-** | **-** | **-** | **-** | **-** | **-** | **-** | **-** |
| DXP | | - | - | - | - | - | - | - | - | - |
| FEP+IBP | | 2 | 0.125 | 8 | 8 | 2 | 4 | 256 | 128 | 256 |
| FEP+ASA | | 2 | 0.25 | 64 | 64 | 16 | 32 | 128 | 64 | 64 |
| FEP+DXP | | 4 | 0.5 | 1024 | 1024 | 64 | 256 | 1024 | 512 | 1024 |
| **FEP** | **MBEC** | **512** | **256** | **2048** | **2048** | **1024** | **2048** | **4096** | **4096** | **4096** |
| IBP | | - | - | - | - | - | - | - | - | - |
| ASA | | - | - | - | - | - | - | - | - | - |
| DXP | | - | - | - | - | - | - | - | - | - |
| FEP+IBP | | 256 | 128 | 512 | 256 | 512 | 1024 | 2048 | 2048 | 2048 |
| FEP+ASA | | 512 | 256 | 1024 | 1024 | 1024 | 2048 | 2048 | 2048 | 2048 |
| FEP+DXP | | 1024 | 1024 | 4096 | 4096 | 2048 | 4096 | 4096 | 4096 | 4096 |

**MIC:** Minimum Inhibitory Concentration**, MBIC:** Minimal Biofilm Inhibitory Concentration**, MBEC:** Minimal Biofilm Eradication Concentration**, FEP:** Meropenem**, DXP:** Dexamethasone sodium phosphate**, ASA:** Aspirin (Acetylsalicylic acid)**, IBP:** Ibuprofen. **-**: No antibacterial or anti-biofilm effects were observed.
